# Association between the *ACTN3* R577X polymorphism and skeletal muscle mass: a systematic review and meta-analysis

**DOI:** 10.1186/s40101-026-00439-5

**Published:** 2026-07-27

**Authors:** Sakura Hattori, Mika Kamiya-Saito, Naoki Kikuchi, Koichi Nakazato

**Affiliations:** 1 Graduate School of Health and Sport Science, Nippon Sport Science University, Tokyo, Japan; 2 Research Institute for Sport Science, Nippon Sport Science University, Tokyo, Japan

**Keywords:** ACTN3, α-actinin-3, ACTN3 R577X polymorphism, Skeletal muscle mass, Sarcopenia

## Abstract

**Background:**

The *ACTN3* R577X polymorphism is one of the genetic factors influencing skeletal muscle mass. Although several studies have reported that the X allele of the *ACTN3* R577X polymorphism is associated with reduced muscle mass, others have found no significant relationship between genotype and muscle mass. These inconsistencies may be explained by differences in background characteristics of the study populations, such as sex, age, ethnicity, and measurement site. This systematic review and meta-analysis aimed to clarify the association between the *ACTN3* R577X polymorphism and skeletal muscle mass. In addition, we investigated whether the effect of the *ACTN3* gene is modified by population background.

**Main body:**

A total of 34 cohorts (9,496 participants) derived from 24 studies were included in the meta-analysis. In the overall analysis under the dominant model for the X allele (RR vs. RX + XX), the *ACTN3* genotype showed a significant effect on skeletal muscle mass (SMD [95% CI] = − 0.08 [− 0.15 to − 0.02], *p* = 0.014), with the RX + XX group exhibiting lower skeletal muscle mass compared with the RR group. In contrast, the recessive model (RR + RX vs. XX) did not yield a statistically significant effect (SMD [95% CI] = − 0.02 [− 0.12 to 0.07], *p* = 0.616). Under the additive model, a trend toward an effect was observed (per-allele; β [95% CI] = − 0.05 [− 0.10 to 0.01], *p* = 0.078). Moderate heterogeneity was observed across all models (dominant: *I*^2^ = 38.8%; recessive: *I*^2^ = 55.8%; additive: *I*^2^ = 52.5%). No significant subgroup effects were observed for sex, age, or ethnicity. Meta-regression for measurement site indicated that the association became more pronounced as lower-limb specificity increased, although residual heterogeneity remained.

**Conclusion:**

The *ACTN3* R577X polymorphism showed a small association with skeletal muscle mass under the X-allele dominant model, suggesting that this association may be detectable even in heterozygous individuals. Although measurement site may modulate this association, none of the examined factors substantially explained the observed heterogeneity when considered individually. The remaining variability may reflect combinations of background characteristics that were not fully captured by single-factor analyses. Further studies accounting for background characteristics are warranted to clarify the genetic contribution of *ACTN3* R577X to skeletal muscle mass.

**Supplementary Information:**

The online version contains supplementary material available at https://doi.org/10.1186/s40101-026-00439-5.

## Introduction

Sarcopenia is recognized not merely as a decline in musculoskeletal function but as a risk factor for various diseases [1]. Skeletal muscle serves multiple critical physiological roles beyond mechanical support, functioning as a glucose metabolic tissue [1, 2], an endocrine organ [3], an energy reservoir [4, 5], and a site for drug metabolism [6–8]. Therefore, maintaining adequate skeletal muscle mass is essential for preventing age-related sarcopenia and frailty, reducing the risk of lifestyle-related diseases, and improving clinical outcomes across diverse medical conditions.

The importance of genetic determinants in individual differences in skeletal muscle mass and sarcopenia has been increasingly recognized in recent years. Twin and family studies have reported that heritability estimates for muscle mass–related traits, including lean body mass, limb circumference, and cross-sectional area, range from approximately 56% to over 80%, depending on adjustment factors [9–13]. Elucidating the mechanisms underlying this diversity in muscle mass may enable personalized risk management and the prevention of sarcopenia. Despite the identification of numerous associated genes [14], the full landscape of genetic factors determining skeletal muscle mass remains unclear.

The *ACTN3* rs1815739 polymorphism is one of the genetic factors suggested to be associated with skeletal muscle mass. This variant leads to the replacement of an arginine residue with a premature stop codon at position 577 (R577X), a nonsense allele. *ACTN3* knockout (KO) mice, which model the R577X, have demonstrated that deficiency of α-actinin-3, encoded by the *ACTN3* gene, reduces skeletal muscle mass [15–18]. Although these animal studies consistently support the association between *ACTN3* genotype and muscle mass, findings in humans remain inconsistent. Zempo et al. (2010) reported that RR + RX genotype exhibited greater muscle mass than the XX genotype [19], whereas a large cohort study by Delmonico et al. (2008) found no significant genotype-related differences [20].

These inconsistencies may be attributable not only to limitations in statistical power due to small sample sizes, but also to differences in population characteristics, such as age [21, 22] and sex [21, 23, 24], and in assessment methods, including the measurement site from which skeletal muscle mass was evaluated [25, 26]. In addition, given that the *ACTN3* polymorphism has been suggested to have undergone evolutionary selection pressures [15, 27, 28], it is plausible that differences in genetic background across populations may further modify the phenotypic effects of this variant. Thus, to elucidate the physiological significance of the *ACTN3* polymorphism, it is necessary to adopt a multifaceted perspective that considers not only the factors previously reported in the literature but also population-specific genetic contexts that may influence its effects.

Accordingly, the present study aimed to systematically evaluate the association between the *ACTN3* R577X polymorphism and skeletal muscle mass and to gain insights into factors that may modulate this association by conducting a meta-analysis along with subgroup analyses based on potential effect modifiers.

## Method

### Eligibility criteria

Only studies that investigated the association between the *ACTN3* R577X polymorphism and skeletal muscle mass were considered eligible for inclusion. Specifically, studies were included if they (1) examined the association between the *ACTN3* R577X polymorphism and skeletal muscle mass; (2) enrolled human participants, with no restrictions on age, sex, physical activity level, or athletic status; (3) reported quantitative measures of skeletal muscle mass, such as lean body mass (LBM), fat-free mass (FFM), muscle cross-sectional area (CSA), or muscle volume; and (4) were published as full-text articles in the English language.

Studies were excluded if they (1) were review articles, meta-analyses, animal studies, or in vitro experiments; (2) did not provide separate data for the *ACTN3* RX genotype in addition to the RR and XX genotypes; (3) lacked sufficient statistical data to calculate effect sizes (i.e., mean and standard deviation of skeletal muscle mass for each *ACTN3* R577X genotype); or (4) contained participant data that overlapped with a previously included publication.

### Search strategy

The PubMed, Web of Science, and SPORTDiscus databases were searched up to November 2025 using the following search string: (ACTN3 OR actinin3 OR “alpha actinin3”) AND (“muscle mass” OR “lean body mass” OR “muscle volume” OR “cross sectional area” OR “Muscular Atrophy” OR “Sarcopenia”).

### Study records

All search results were exported to Rayyan [29] and Microsoft Excel (Microsoft Corp.) for recordkeeping and data management. The Excel spreadsheets were used to document the search records and organize the extracted data. Full-text articles and associated datasets were stored in a shared Dropbox folder, which was accessible to all review authors.

Two authors independently screened the titles and abstracts of all retrieved records to identify potentially eligible studies. The full texts of the remaining articles were subsequently assessed for eligibility. Any disagreements between reviewers were resolved through discussion. In addition, further relevant studies were identified by screening the reference lists of the included articles. Reasons for study exclusion at the full-text stage were systematically documented.

Data were independently extracted by two authors using a standardized extraction form. The following information was collected from each study: PubMed ID, article title, authors, journal name, year of publication, skeletal muscle mass data (mean, standard deviation [SD] or standard error [SE], and sample size for each genotype), participant characteristics (sex, age, country, height, weight), phenotype information, and methodological details. For studies involving training interventions, skeletal muscle mass data at baseline before the intervention were extracted. When necessary, the corresponding study authors were contacted to obtain summary statistics required for the meta-analysis.

### Quality assessment

The methodological quality and risk of bias of the included studies were assessed using the Risk of Bias Assessment Tool for Non-randomized Studies, version 2 (RoBANS-2) [30], which is specifically designed to evaluate cross-sectional studies. Each study included in the meta-analysis was independently assessed for risk of bias by two reviewers. The assessment was performed separately for each participant group analyzed in the meta-analysis, and any disagreements were resolved through discussion. An overall risk-of-bias judgment for each study was determined according to the following predefined rules: a study was judged to have a high risk of bias if at least one domain was rated as High; a low risk of bias if all domains were rated as Low or if only one domain was rated as unclear; and an unclear risk of bias in all other cases.

Potential publication bias was evaluated by visually inspecting funnel plots and statistically tested using Egger’s regression test [31].

### Statistical analysis

Genotype effects were evaluated under three commonly used genetic models: dominant, recessive, and additive for the X allele. In the dominant model (RR vs RX + XX) and the recessive model (RR + RX vs XX), genotype groups were first collapsed according to the assumed inheritance pattern, and the standardized mean difference (SMD) was then calculated based on the resulting two-group comparison. Hedges’ *g* was used as the SMD and was calculated from the mean, standard deviation, and sample size of each group to account for potential small-sample bias in individual studies [30]. In the additive model, genotypes were coded as 0 (RR), 1 (RX), and 2 (XX), and the per-allele effect size was calculated as the regression coefficient (β). All effect sizes were oriented so that more negative values indicated lower skeletal muscle mass in association with the X allele.

A random-effects model was used to synthesize the data, as heterogeneity among studies was anticipated. The Knapp-Hartung adjustment was applied to calculate the 95% CI for the pooled effect size, as this method provides a more robust estimate in the presence of heterogeneity [32]. Statistical heterogeneity was evaluated with Cochran’s *Q* test and quantified using the *I*^2^ statistic. Following the recommendations of Higgins et al. (2002), *I*^2^ values of 25%, 50%, and 75% were interpreted as representing low, moderate, and high levels of heterogeneity, respectively [33].

Furthermore, to assess the robustness of the pooled results, a leave-one-out sensitivity analysis was performed. This involved iteratively removing one study at a time and recalculating the overall effect size to ensure that the results were not unduly influenced by any single cohort.

To explore potential sources of heterogeneity, we conducted subgroup analyses based on pre-specified study characteristics: sex, age, ethnicity, and measurement site. The age variable was dichotomized into two categories, defined as young (< 60 years old) and old (≥ 60 years old). The ethnicity was determined for each study based on participant information and the country where the experiment was conducted. The statistical significance of differences between subgroups was assessed using Cochran’s* Q* test, and the degree of heterogeneity was quantified using the *I*^2^ statistic [34]. For each subgroup analysis, datasets with missing data for the subgroup-defining variables were excluded on an analysis-specific basis.

To examine whether the effect size under the additive model varied according to measurement site, measurement-site categories were coded as an ordinal moderator reflecting increasing lower-limb specificity. Whole-body, four-limb, and lower-limb categories were assigned values of 0, 1, and 2, respectively. A meta-regression analysis was then performed with this ordinal variable entered as a continuous moderator. The model was fitted using restricted maximum likelihood estimation, and statistical inference for the moderator was based on the Knapp–Hartung adjustment. The proportion of heterogeneity explained by the moderator was expressed as *R*^2^_*_.

All analyses were performed using R (version 4.5.1) with the meta package (version 8.2.0) and metafor package (version 4.8.0). A two-sided *p* value < 0.05 was considered statistically significant for all analyses.

## Result

### Study selection

**Fig. 1:**
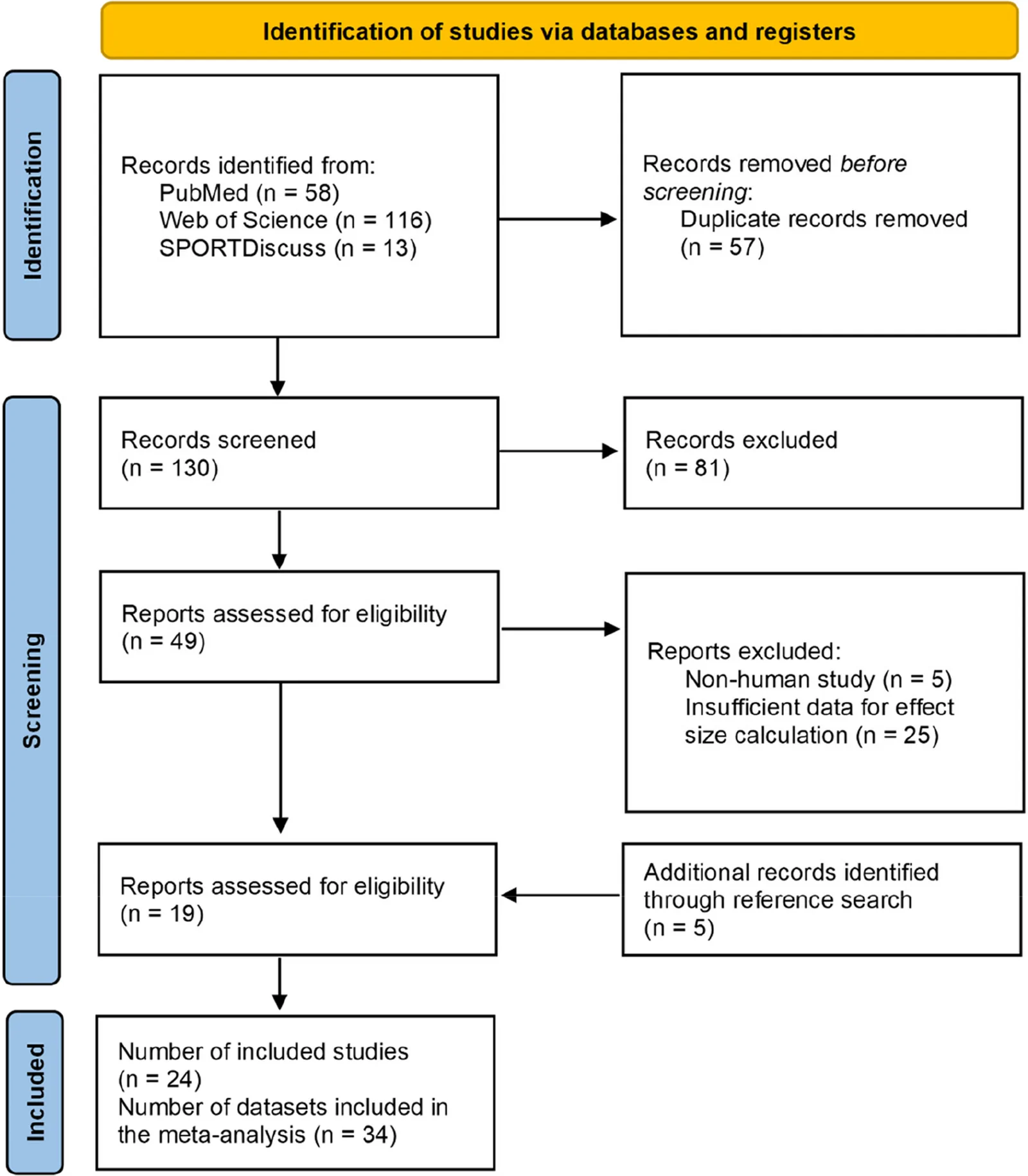
PRISMA flow chart of the study selection procedure PRISMA flow diagram (adapted from the PRISMA 2020 template). Original from Page MJ et al. (2021) [35], licensed under CC BY 4.0 (https://creativecommons.org/licenses/by/4.0/)

The study selection process is summarized in Fig. 1. Our initial database search yielded 187 potential articles. After removing 57 duplicates, 130 articles were screened. After excluding 81 records based on title and abstract review, we assessed 49 articles at the full-text level. In addition to the 19 studies identified from the primary search, five more were included through a reference search. Ultimately, 24 studies satisfied the eligibility criteria, from which 34 independent datasets were extracted and included in the meta-analysis.

### Study characteristics

A total of 24 studies were included, with sample sizes ranging from 32 to 1152 participants per cohort. The cohorts comprised both young (< 60 years) and old (≧ 60 years), and included male, female, or both sexes. Studies were conducted across East Asia, Europe, and admixed American groups. Outcomes included muscle volume, cross-sectional area, and fat-free mass, assessed using dual-energy X-ray absorptiometry (DEXA), magnetic resonance imaging (MRI), Bioelectrical Impedance Analysis (BIA), or skinfold methods, with measurements reported for either appendicular or whole-body compartments. The detailed characteristics of the included studies are presented in Table 1.

**Table 1:** Characteristics of studies were included in meta-analysis

Study	Sex	*N*	Age	Age group	Country	Ethnicity	Outcome	Unit	Body region
Boshnjaku et al. 2021 [36]	B	282	67.6	Old	Kosovan	European	FFM (BIA)	kg	Whole
Cho et al. 2017 [37]	B	332	≧ 65	Old	Korea	East Asian	MV (DEXA)	kg/m^2^	Four limbs
Kiuchi et al. 2021 [38]	B	64	74.5	Old	Japan	East Asian	MV (MRI)	cm^3^	Lower limbs
Zempo et al. 2010 [26]	F	109	64.1	Old	Japan	East Asian	CSA (MRI)	cm^2^	Lower limbs
Broos et al. 2015 [39]	M	266	20.9	Young	Belgium	European	CSA (Skinfold)	cm^2^	Lower limbs
Lima et al. 2011 [40]	F	234	66.7	Old	Brazil	Admixed American	FFM (BIA)	kg	Whole
Min et al. 2016 [41]	F	68	67.4	Old	Korea	East Asian	MV (DEXA)	kg	Whole
Zempo et al. 2011a [22]	F	82	50.6	Young	Japan	East Asian	CSA (MRI)	cm^2^	Lower limbs
Zempo et al. 2011b [22]	F	80	66.8	Old	Japan	East Asian	CSA (MRI)	cm^2^	Lower limbs
Erskine et al. 2014 [42]	M	51	20.3	Young	(Caucacian)	European	MV (MRI)	cm^3^	Lower limbs
Urzi et al. 2021a [43]	M	67	65–78	Old	Slovenia	European	FFM (BIA)	kg/m^2^	Whole
Urzi et al. 2021b [43]	F	123	65–78	Old	Slovenia	European	FFM (BIA)	kg/m^2^	Whole
Delmonico et al. 2008a [20]	M	726	73.9	Old	America (White)	European	MV (DEXA)	kg	Lower limbs
Delmonico et al. 2008b [20]	F	641	73.5	Old	America (White)	European	MV (DEXA)	kg	Lower limbs
Delmonico et al. 2008c [20]	M	1152	73.7	Old	America (White)	European	MV (DEXA)	kg	Lower limbs
Clarkson et al. 2005a [44]	M	247	24.9	Young	N/A	N/A	CSA (MRI)	cm^2^	Upper limbs
Clarkson et al. 2005b [44]	F	355	24.3	Young	N/A	N/A	CSA (MRI)	cm^2^	Upper limbs
Vincent et al. 2007 [45]	M	90	21.7	Young	Belgium	European	FFM (BIA)	kg	Whole
Walsh et al. 2008a [46]	M	416	22–90	N/A	N/A	N/A	MV (DEXA)	kg	Whole
Walsh et al. 2008b [46]	F	394	22–90	N/A	N/A	N/A	MV (DEXA)	kg	Whole
Norman et al. 2009a [47]	M	61	25.3	Young	Sweden	European	FFM (Skinfold)	kg	Whole
Norman et al. 2009b [47]	F	59	24	Young	Sweden	European	FFM (Skinfold)	kg	Whole
McCauley et al. 2010 [48]	M	100	65	Old	United Kingdom	European	MV (DEXA)	kg	Whole
Hanson et al. 2010 [49]	B	57	22	Young	N/A	N/A	FFM (Skinfold)	kg	Whole
Abudurezake et al., 2025a [50]	M	603	72.9	Old	Japan	East Asian	FFM (BIA)	kg	Four limbs
Abudurezake et al., 2025b [50]	F	881	73	Old	Japan	East Asian	FFM (BIA)	kg	Four limbs
Iwasaka et al., 2024a [51]	M	404	61.3	Old	Japan	East Asian	FFM (BIA)	kg/m^2^	Whole
Iwasaka et al., 2024b [51]	F	619	60.8	Old	Japan	East Asian	FFM (BIA)	kg/m^2^	Whole
Silva et al., 2024a [52]	B	32	69.5	Old	Brazil	Admixed American	N/A	kg/m^2^	Whole
Silva et al., 2024b [52]	B	39	63	Old	Brazil	Admixed American	N/A	kg/m^2^	Whole
Shin et al., 2024 [53]	N/A	81	29.3	Young	Korea	East Asian	FFM (BIA)	kg	Whole
Taniguchi et al., 2022 [54]	B	295	73.2	Old	Japan	East Asian	FFM (BIA)	kg/m^2^	Four limbs
Güereca-Arvizuo J et al., 2022 [55]	M	35	18–35	Young	Mexico	Admixed American	FFM (BIA)	kg	Whole
Shang et al., 2012 [56]	M	452	21.0	Young	China	East Asian	FFM (BIA)	kg	Whole

Age groups were defined as young (< 60 years) and older (≥ 60 years). Parentheses (e.g., “Caucasian”) denote studies in which the country was not reported and only race was provided. Parentheses in the Outcome column indicate the measurement method. *Abbreviations*: *B* both sex, *F* female, *M* male, *FFM* free-fat mass, *MV* muscle volume, *CSA* cross-sectional area, *BIA* bioelectrical impedance analysis, *DEXA* dual-energy X-ray absorptiometry, *MRI* magnetic resonance imaging

### Publication bias and risk-of-bias assessment

The results of the risk-of-bias assessment are summarized as a traffic-light plot in Additional file 1. Overall, 13 analyses were judged to have a high risk of bias, 14 were judged as low risk, and 7 were unclear. The primary domains contributing to a High risk of bias were Confounders (D3), followed by Comparability of the target group (D1), Blinding of assessors (D5), Outcome assessment (D6), and Incomplete outcome data (D7). The risk of bias summary was presented with consideration of study weights from the random-effects model used in the meta-analysis (Additional file 1). Using this weighting approach, studies with a high risk of bias accounted for approximately 25% of the evidence across the dominant, recessive, and additive models.

Funnel plots for all genotype models are shown in Additional file 2. Egger’s test indicated no significant publication bias in any of the genotype models (dominant: *p* = 0.174, recessive: *p* = 0.910, additive: *p* = 0.290).

### Overall effect of the genotype

**Fig. 2:**
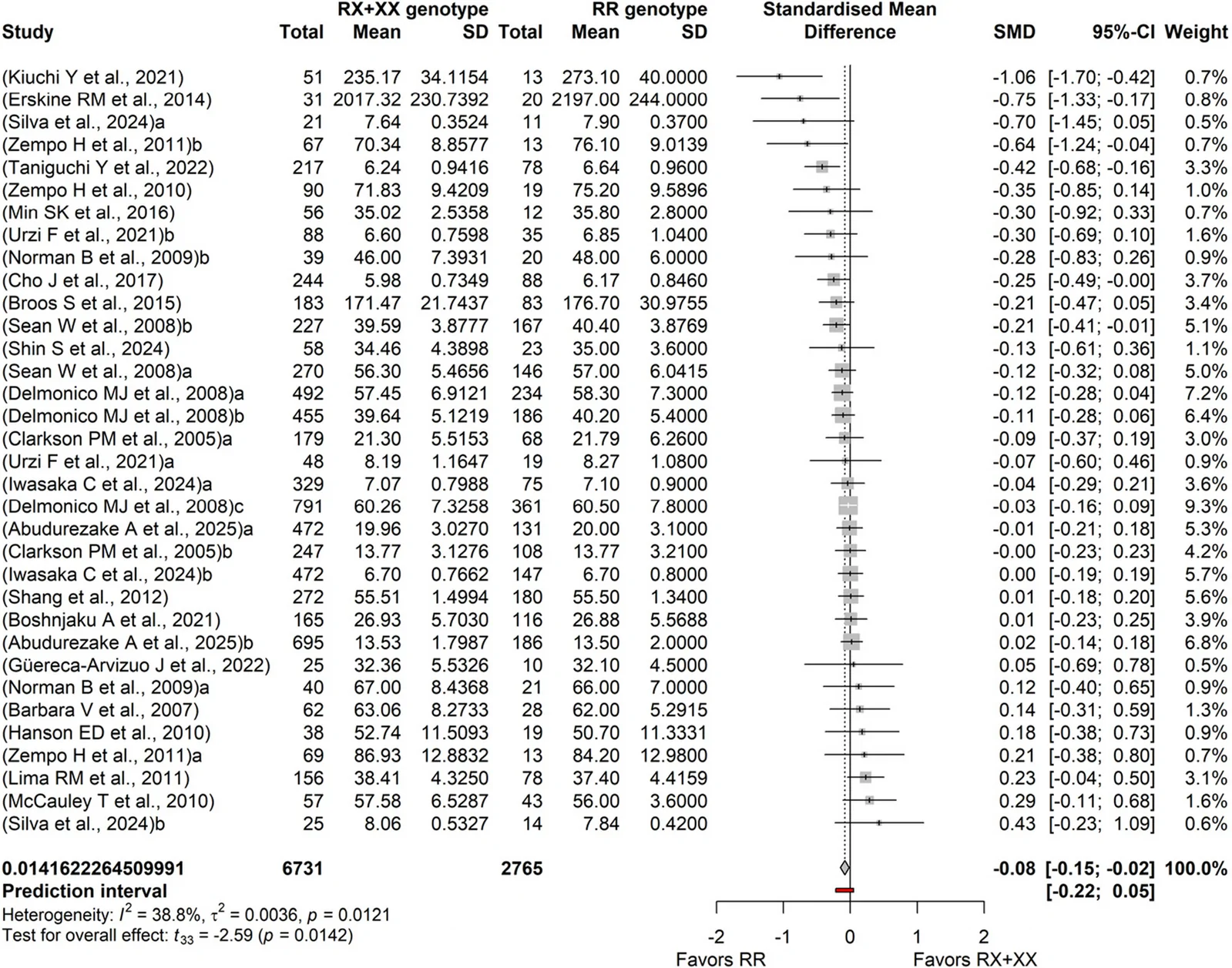
Forest plot comparing skeletal muscle mass between the RR and RX + XX (dominant model) Each square represents the standardized mean difference (SMD) for an individual study, with the horizontal lines indicating 95% confidence intervals (CI). The size of each square is proportional to the study weight under the random-effects model. The diamond at the bottom represents the overall pooled estimate and its 95% CI. Heterogeneity was assessed using the *I*^2^ statistic, with an *I*^2^ value of 38.8%, indicating moderate heterogeneity. The *p*-value for heterogeneity was calculated using Cochran’s *Q* test (*p* = 0.0121). Positive SMD values favor the RX + XX genotype, whereas negative values favor the RR genotype

**Fig. 3:**
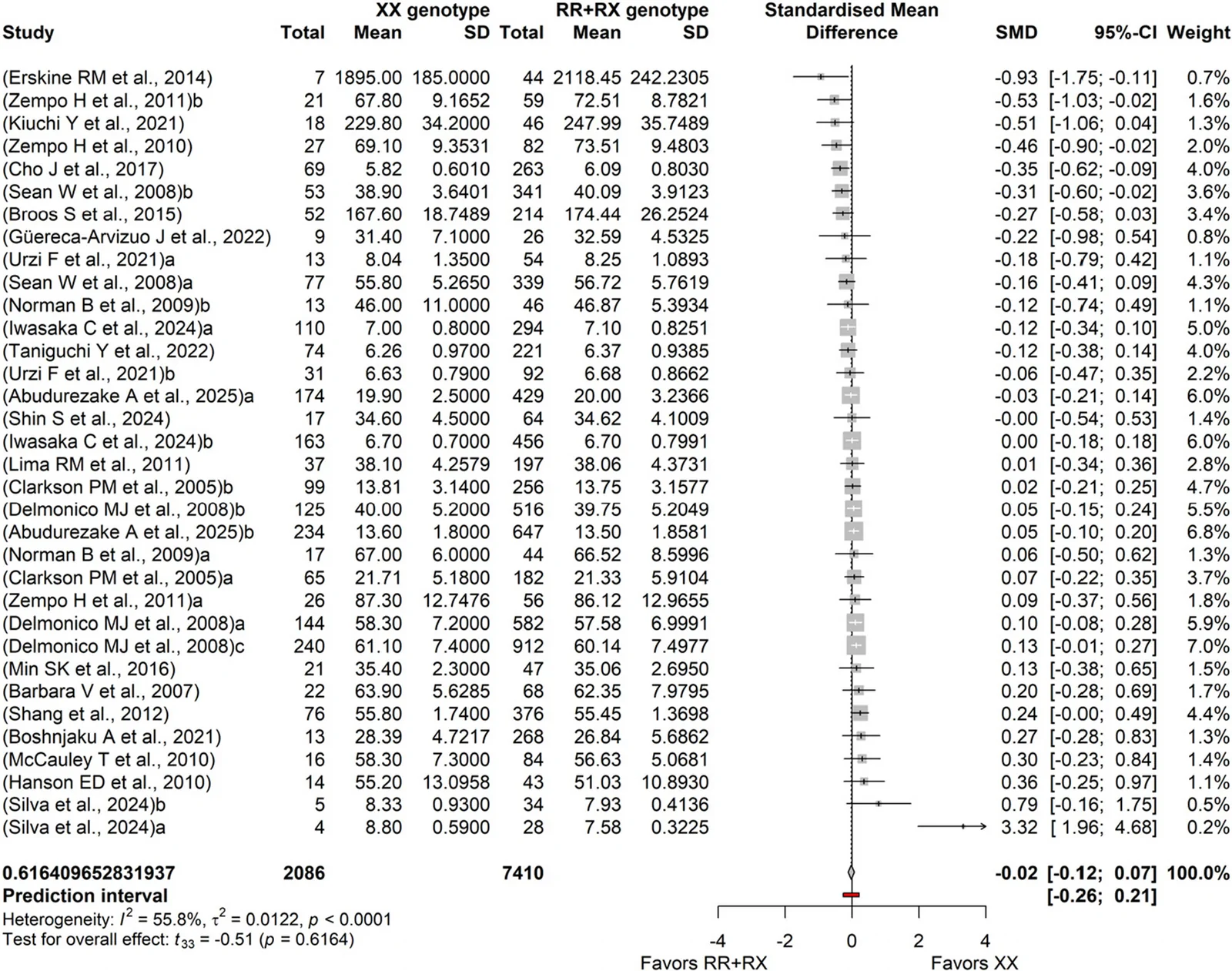
Forest plot comparing skeletal muscle mass between the RR + RX vs XX (recessive model). Each square represents the standardized mean difference (SMD) for an individual study, with the horizontal lines indicating 95% confidence intervals (CI). The size of each square is proportional to the study weight under the random-effects model. The diamond at the bottom represents the overall pooled estimate and its 95% CI. Heterogeneity was assessed using the *I*^2^ statistic, with an *I*^2^ value of 55.8%, indicating moderate heterogeneity. The *p*-value for heterogeneity was calculated using Cochran’s *Q* test (*p* < 0.0001). Positive SMD values favor the XX genotype, whereas negative values favor the RR + RX genotype

**Fig. 4:**
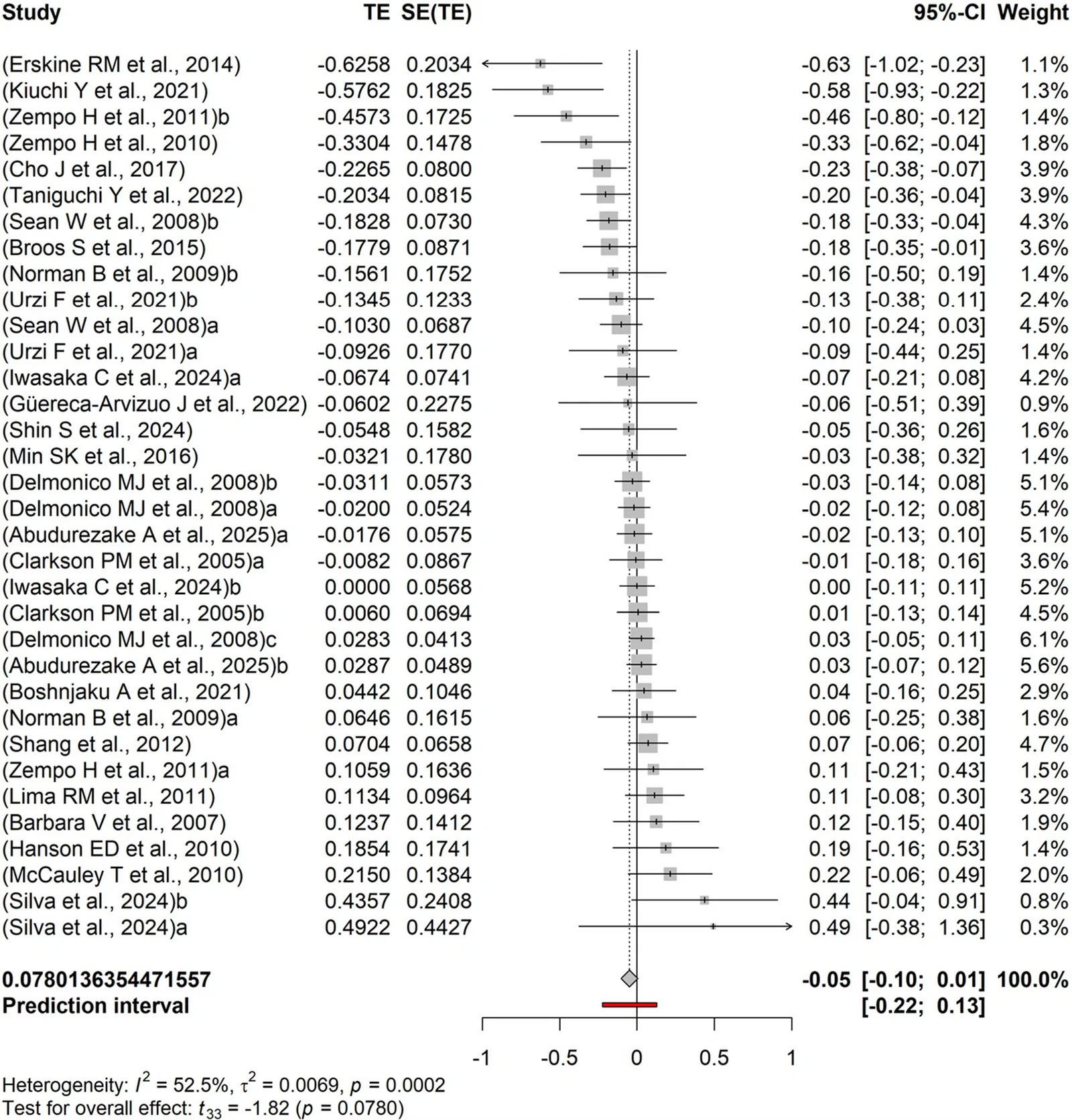
Forest plot comparing skeletal muscle mass across *ACTN3* genotypes (additive model). Each square represents the regression coefficient (β) for an individual study, with the horizontal lines indicating 95% confidence intervals (CIs). The size of each square is proportional to the study weight under the random-effects model. The diamond at the bottom represents the overall pooled estimate and its 95% CI. Heterogeneity was assessed using the *I*^2^ statistic, with an *I*^2^ value of 52.5%, indicating moderate to substantial heterogeneity. The *p*-value for heterogeneity was calculated using Cochran’s *Q* test (*p* = 0.0002). Negative β values indicate a greater muscle mass associated with the R allele

Under the X allele dominant model, a statistically significant association between the *ACTN3* genotype and skeletal muscle mass was observed, with the combined RX + XX genotype group exhibiting lower skeletal muscle mass compared with the RR genotype group (Fig. 2; SMD = − 0.08 [95% CI − 0.15 to − 0.02], *p* = 0.014). In contrast, the pooled analysis under the recessive model did not show a significant association between *ACTN3* genotype and skeletal muscle mass (Fig. 3; SMD = − 0.02 [95% CI − 0.12 to 0.06], *p* = 0.616). Under the additive model, there was a trend toward an association (Fig. 4; β = − 0.05 [95% CI − 0.10 to 0.00], *p* = 0.078). All model demonstrated moderate heterogeneity (dominant: *I*^2^ 38.8%; Cochran’s *Q* *p* = 0.014, recessive: *I*^2^ 55.8%; Cochran’s *Q* *p* < 0.001, additive: *I*^2^ 52.5%; Cochran’s *Q* *p* < 0.001). Leave-one-out sensitivity analyses indicated that the results were robust, with detailed results presented in Additional file 3.

### Subgroup analysis

We conducted subgroup analyses to explore potential sources of heterogeneity according to sex, age group, ethnicity, and measurement site across all three genetic models. The results of the subgroup analysis are presented in Table 2. In the additive model, there was a trend toward differences in effect size according to measurement site (between-subgroup *I*^2^ = 60.18%, *p* = 0.08; whole body: β = − 0.01 [95% CI − 0.07 to 0.06]; four limbs: β = − 0.09 [95% CI − 0.30 to 0.11]; lower limbs: β = − 0.19 [95% CI − 0.39 to 0.01]).

**Table 2:** Subgroup analysis results across sex, age, ethnicity, and body region groups

Model	Subgroup	*k*	n.e	n.c	ES (95% CI)	*I*^2^	*p*-value	Between *Q* (*p*-value)	Between *I*^2^
Dominant	female	12	2661	984	− 0.07 (− 0.19 to 0.04)	33.86	0.20	0.06 (0.81)	0
	male	14	3251	1419	− 0.06 (− 0.13 to 0.01)	0.00	0.10		
	Old	20	4991	1859	− 0.10 (− 0.21 to 0.01)	53.50	0.09	0.35 (0.55)	0
	Young	12	1243	593	− 0.05 (− 0.16 to 0.06)	0.00	0.33		
	EUR	12	2451	1166	− 0.08 (− 0.17 to 0.01)	21.36	0.08	0.64 (0.42)	0
	EAS	13	3092	978	− 0.14 (− 0.30 to 0.01)	55.33	0.07		
	whole	19	2448	1164	− 0.03 (− 0.11 to 0.05)	10.09	0.44	3.97 (0.14)	49.66
	four_limbs	4	1628	483	− 0.15 (− 0.47 to 0.18)	70.22	0.25		
	lower_limbs	9	2229	942	− 0.24 (− 0.48 to 0.01)	60.32	0.06		
Recessive	female	12	850	2795	− 0.03 (− 0.13 to 0.07)	20.32	0.58	0.22 (0.64)	0
	male	14	1022	3648	0.01 (− 0.11 to 0.12)	39.53	0.90		
	Old	20	1539	5311	− 0.02 (− 0.15 to 0.12)	64.82	0.81	0.16 (0.69)	0
	Young	12	417	1419	0.02 (− 0.13 to 0.17)	23.93	0.75		
	EUR	12	693	2924	0.05 (− 0.06 to 0.16)	23.27	0.35	2.63 (0.11)	61.9
	EAS	13	1030	3040	− 0.08 (− 0.21 to 0.05)	49.13	0.22		
	whole	19	711	2901	0.03 (− 0.14 to 0.20)	56.99	0.71	2.11 (0.35)	5.29
	four_limbs	4	551	1560	− 0.09 (− 0.36 to 0.18)	57.83	0.38		
	lower_limbs	9	660	2511	− 0.15 (− 0.41 to 0.10)	67.29	0.20		
Additive	female	12	NA	NA	− 0.05 (− 0.14 to 0.04)	46.98	0.21	0.45 (0.50)	0
	male	14	NA	NA	− 0.02 (− 0.08 to 0.04)	39.98	0.51		
	Old	20	NA	NA	− 0.05 (− 0.13 to 0.03)	58.38	0.20	0.27 (0.61)	0
	Young	12	NA	NA	− 0.02 (− 0.12 to 0.08)	39.30	0.68		
	EUR	12	NA	NA	− 0.03 (− 0.11 to 0.06)	44.33	0.51	1.47 (0.23)	31.9
	EAS	13	NA	NA	− 0.10 (− 0.21 to 0.01)	64.59	0.07		
	whole	19	NA	NA	− 0.01 (− 0.07 to 0.06)	22.61	0.87	5.02 (0.08)	60.18
	four_limbs	4	NA	NA	− 0.09 (− 0.30 to 0.11)	73.34	0.25		
	lower_limbs	9	NA	NA	− 0.19 (− 0.39 to 0.01)	75.45	0.06		

The column headings represent the following: *k* is the number of included studies in the subgroup analysis. *n.e* and *n.c* represent the sample sizes for the experimental group (RX + XX genotype) and reference group (RR genotype), respectively. ES (95% CI) denotes the pooled effect size and its 95% confidence interval; this represents the standardized mean difference (SMD) for group comparisons or the beta coefficient (β) for the additive model (indicating the effect per copy of the minor allele). Positive SMD values favor the reference group, whereas negative values favor the experimental group. *I*^2^ is the *I*-squared statistic for within-subgroup heterogeneity, reported as a percentage. The *p*-value indicates the statistical significance of the each subgroup’s pooled effect. Between *Q* (*p*-value), and between *I*^2^ indicate statistics for differences between subgroups

**Fig. 5:**
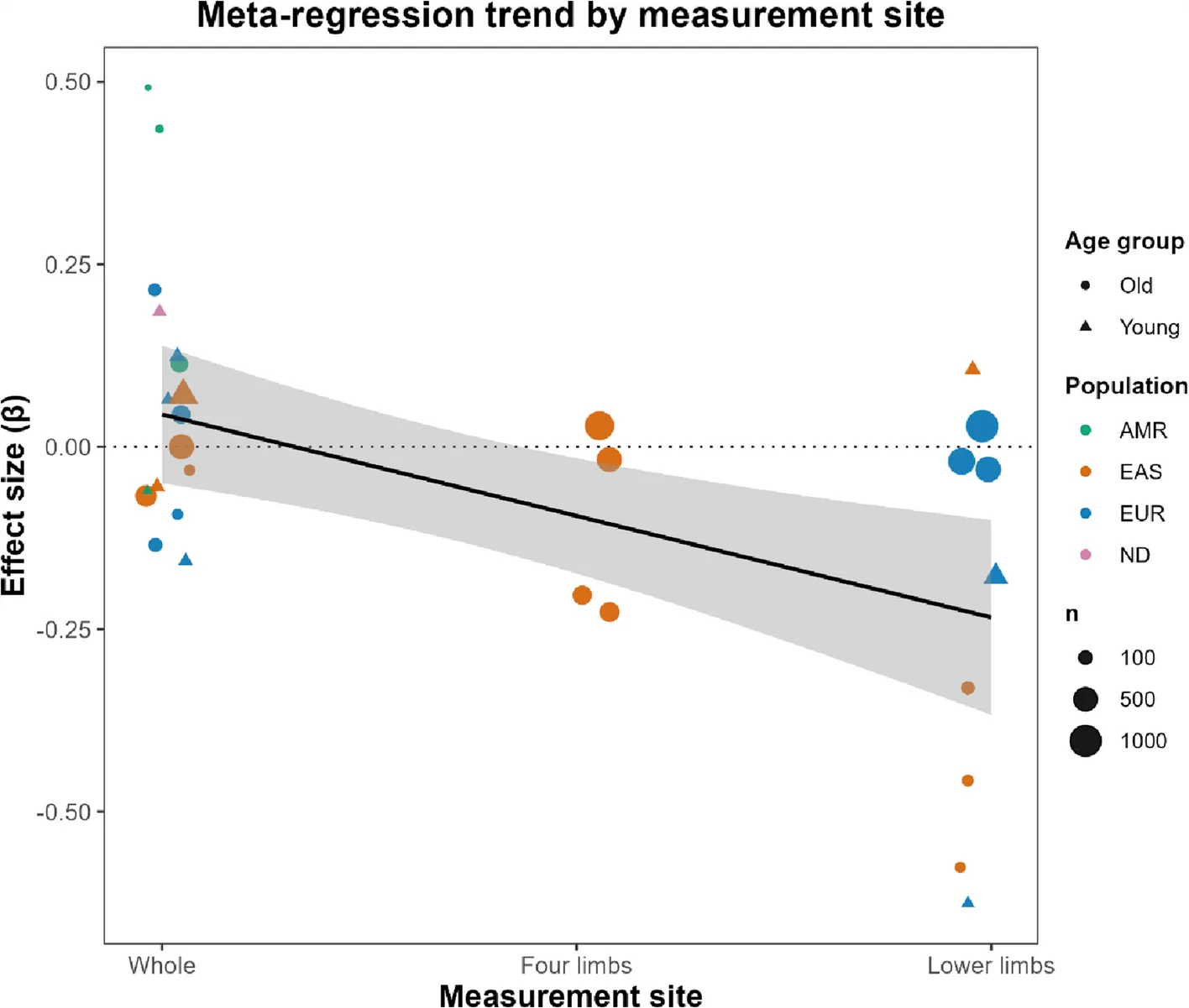
Meta-regression of effect size according to measurement site. Measurement sites were coded as an ordinal variable reflecting increasing lower-limb specificity: whole body (0), four limbs (**1**), and lower limbs (**2**). Each point represents an independent dataset. Point size indicates sample size, color indicates ethnicity group, and shape indicates age group. The solid black line represents the fitted meta-regression line for measurement site, and the gray shaded area represents its 95% confidence interval. More negative effect sizes indicate a stronger association of the *ACTN3 *R577X X allele with lower skeletal muscle mass

Because the measurement-site categories could be interpreted as reflecting increasing lower-limb specificity, whole body, four limbs, and lower limbs were coded as an ordinal variable with values of 0, 1, and 2, respectively. To examine whether differences in effect size across measurement sites followed an ordinal trend, we performed a meta-regression analysis using this ordinal variable. The meta-regression analysis showed a negative association between the ordinal measurement-site variable and effect size (Fig. 5; β = − 0.072 [95% CI − 0.140 to − 0.004], *p* = 0.039), indicating that the X allele was more strongly associated with lower muscle mass as the measurement site became more specific to the lower limbs. However, this linear trend did not explain the overall heterogeneity (*R*^2^_*_, amount of heterogeneity accounted for = 0.00%). Therefore, we visually explored whether the trend across measurement sites coincided with other study-level characteristics, such as ethnicity and age group. Visual inspection of the plot suggested that some datasets involving EAS populations, older participants, and lower-limb measurements showed more negative effect sizes, as indicated by the orange circles (Fig. 5).

## Discussion

This systematic review and meta-analysis investigated the association between the *ACTN3* R577X polymorphism and skeletal muscle mass. The overall effect on the dominant model was statistically significant, with the combined RX + XX genotypes exhibiting lower skeletal muscle mass compared with the RR genotype. Because moderate heterogeneity was observed across all genetic models, we examined whether the effects of the *ACTN3* polymorphism differed according to population characteristics and assessment methods. As a result, a trend toward a significant subgroup effect was observed among measurement-site categories. Meta-regression further suggested that the association of the X allele with lower skeletal muscle mass became more pronounced with increasing lower-limb specificity.

The findings of a significant association under the dominant model imply that even the heterozygous (RX) state may exhibit partial functional deficiency. Biologically, the *ACTN3* R577X polymorphism is a nonsense variant; individuals with the XX genotype lack α-actinin-3, whereas those with the RX genotype express it. Therefore, this variant is predicted to follow a recessive pattern. However, the observed data suggests that the functional impact may not be strictly recessive, indicating an intermediate phenotype for heterozygotes. Supporting this notion, studies using *Actn3* knockout mice suggest that the heterozygous genotype is associated with reduced muscle mass, accompanied by a significant reduction in muscle fiber diameter [57]. In humans, several studies have reported that the effects of *ACTN3* genotype may be additive. For example, a study comparing 40-m sprint time showed that the association between ACTN3 genotype and sprint performance was consistent with an additive model [58]. In addition, a meta-analysis examining the association between *ACTN3* gene polymorphism and flexibility reported a significant association only under the recessive model [59]. In present study, while the dominant model yielded significant results, the additive model showed only a trend. This pattern may indicate that the additive trend was partly driven by the RX genotype showing a phenotype closer to the XX genotype than to the RR genotype.

One possible mechanism through which the *ACTN3* R577X polymorphism may influence skeletal muscle mass is an increased vulnerability of muscle fiber structure resulting from reduced expression of α-actinin-3. Loss of α-actinin-3 due to the R577X variant is accompanied by compensatory upregulation of α-actinin-2, thereby preserving the total amount of actinin proteins [60]. However, this compensation appears insufficient to fully maintain structural integrity, potentially leading to reduced mechanical stability of muscle fibers. Consistent with this notion, studies in *Actn3* knockout mice have demonstrated greater muscle damage following eccentric contractions [60, 61]. Moreover, even under resting conditions, increased expression of Z-disk–associated proteins and the presence of centrally located nuclei have been observed, suggesting the presence of chronic muscle stress and ongoing remodeling processes [60]. Such persistent structural stress may impair satellite cell regenerative capacity through repeated cycles of muscle damage and regeneration, as has been reported in Duchenne muscular dystrophy, ultimately compromising the maintenance of muscle mass [62]. Therefore, sustained structural fragility resulting from the absence or reduction of α-actinin-3 may promote chronic muscle fiber damage and contribute to decreased skeletal muscle mass via impaired satellite cell–mediated regeneration. In addition, increased expression of Z-disk–related proteins, such as desmin and ZASP, has also been reported in heterozygous mice carrying the RX genotype [57]. This observation is consistent with the present findings, suggesting that the effects of the *ACTN3* polymorphism may already be evident in individuals carrying a single X allele.

Because previous studies have suggested that the effects of the *ACTN3* R577X polymorphism may differ between sexes [21, 23, 24], we examined the influence of genotype on skeletal muscle mass in male and female participants using subgroup analysis. However, when the available studies were quantitatively synthesized, no clear sex-specific difference in the genotype effect was observed. A recent study using skeletal muscle from Actn3 knockout mice and human muscle biopsy samples reported that androgen receptor protein levels were lower in both Actn3 knockout mice and individuals with the XX genotype, irrespective of sex [63]. In contrast, studies using androgen receptor knockout mice have shown that the reduction in skeletal muscle mass caused by androgen receptor deficiency is evident in males, but not in females [64, 65]. These findings suggest that the effect mediated by reduced androgen receptor signaling may predominantly operate in males. In females, however, skeletal muscle mass may be more strongly influenced by female sex hormones, including estrogen. Notably, α-actinin-2 has been reported to interact not only with androgen receptor activity but also with estrogen receptor activity [66], suggesting that altered estrogen signaling may also contribute to reduced skeletal muscle mass. Therefore, the absence of a detectable sex difference in the present subgroup analysis may reflect distinct but convergent interactions between *ACTN3* genotype and sex hormone-related pathways in males and females. Further studies are needed to clarify these interactions, particularly the potential involvement of estrogen signaling.

In the subgroup analysis stratified by measurement site, a trend toward a significant subgroup effect was observed under the additive model. When we further examined whether the effect size varied linearly according to increasing lower-limb specificity, the model showed a significant linear fit. Specifically, the association of the X allele with reduced muscle mass became stronger as the measurement site became more specific to the lower limbs, suggesting that the effect of the X allele may be more pronounced in lower-limb measurements. This site-specific tendency may be related to impaired remodeling following the structural damage described above. Lower-limb muscles are repeatedly exposed to weight-bearing mechanical loads and eccentric contractions during daily activities [67, 68]. Therefore, reduced tolerance to mechanical stress caused by α-actinin-3 deficiency may be more likely to manifest over time as differences in damage repair and remodeling responses in lower-limb muscles. Although muscle mass is one of the major determinants of muscle strength, a meta-analysis examining the effect of *ACTN3 *R577X on muscle strength did not show the lower-limb-specific effect observed in the present study [69]. One possible explanation for this discrepancy is that variation in the effect on muscle mass according to measurement site may not have been large enough to be detected as a clear site-specific difference in muscle strength, which is a complex phenotype. Muscle strength is determined not only by muscle mass but also by multiple factors, including muscle fiber-type composition, contractile properties, and neural control. *ACTN3 *R577X, through α-actinin-3 deficiency, has been reported to reduce the proportion of Type IIx fibers, which generate high tension per unit cross-sectional area [24, 45]. Thus, changes in muscle fiber composition and muscle quality may have obscured the influence of site-specific variation in muscle mass when muscle strength was assessed as the outcome.

The linear effect across measurement sites did not explain the overall heterogeneity, and the other subgroup factors also did not show detectable subgroup effects when examined individually. These findings suggest that heterogeneity may reflect unexamined factors or the combined influence of multiple factors. Although the limited number of available datasets prevented us from statistically examining how combinations of multiple factors influence variability in the effect of *ACTN3 *R577X (Fig. 5), which visualizes the study-level categories, suggests that the X allele may be associated with lower lower-limb muscle mass particularly in older East Asian populations. Further studies including diverse background populations are therefore warranted to clarify the influence of such combined factors. In particular, data on lower-limb muscle mass in young East Asian populations remain especially limited, and additional evidence from this group is needed to determine whether the apparent effect of the X allele on lower muscle mass is specific to older East Asian individuals.

In the present meta-analysis, the overall effect sizes were small, indicating that the contribution of the *ACTN3* genotype alone to skeletal muscle mass is limited and unlikely to represent a pathological phenotype associated with a substantially increased risk of sarcopenia. From an alternative perspective, the modestly lower muscle mass associated with the X allele may reflect a potentially rational and adaptive phenotype. Skeletal muscle is a primary site of energy expenditure during physical activity and is considered a metabolically costly tissue [70, 71]. Therefore, a slight reduction in muscle mass that does not impair activities of daily living could theoretically enhance whole-body energy efficiency and confer an advantage for survival. In line with this notion, a study of Spanish centenarians reported a potential contribution of the X allele to longevity [72]. Such an interpretation may provide a plausible evolutionary framework for understanding the signatures of selection at the *ACTN3* locus. Energy conservation is a critical factor for survival and reproduction. Previous studies have suggested that the X allele-associated shift toward a more aerobic muscle metabolism improves energetic efficiency and may have been advantageous during human evolution [15]. The modest reduction in muscle mass observed in the present analysis may be interpreted within a similar evolutionary context. Nevertheless, further research is required to clarify the contribution of the X allele to natural selection.

## Limitation

The number of available studies was limited, with data obtained from a total of 34 cohorts. We were unable to examine subgroup effects related to athlete status, sport participation, exercise habits, or physical activity levels because sufficient data for these factors could not be obtained. In particular, we did not identify any datasets that reported both skeletal muscle mass and *ACTN3 *R577X genotype in athletes. Evaluating the potential modifying effects of physical activity levels, athlete status, and training history on the association between *ACTN3 *R577X and skeletal muscle mass may be important because previous studies have suggested that individuals with the XX genotype may show attenuated training-induced muscle adaptations, potentially through reduced activation of hypertrophy-related pathways such as mTORC1 signaling [73, 74]. In the present study, only baseline skeletal muscle mass was extracted from intervention studies; therefore, genotype-related differences that may emerge after training or in highly trained populations could not be captured.

Furthermore, multiple cohorts were extracted from the same studies. Although we confirmed in the Methods section that there was no overlap of participants, data derived from the same study inevitably share the same study design and data collection methods. Thus, the cohorts cannot be regarded as completely independent, and this may have influenced the pooled estimates. Finally, different measurement methods for skeletal muscle mass were included. The present meta-analysis incorporated data assessed by fat-free mass, muscle volume, and cross-sectional area. To integrate these heterogeneous measures, standardized mean differences were calculated based on the standard deviation of each dataset. Therefore, the results should be interpreted with caution, because the effect size reflects relative differences standardized by variability among studies, rather than absolute differences in skeletal muscle mass.

## Conclusion

This meta-analysis suggests that the *ACTN3 *R577X polymorphism is associated with skeletal muscle mass and that this effect may be detectable even in heterozygous individuals. However, the magnitude of this association was very small. Differences in measurement sites were suggested to modulate the effect of *ACTN3 *R577X on skeletal muscle mass; however, measurement site alone did not substantially explain the observed heterogeneity. Similarly, none of the other factors examined in the present study, including sex, age, and ethnicity, substantially explained the variability in the effect of *ACTN3 *R577X when assessed as a single factor. The remaining heterogeneity may reflect the combined influence of multiple background characteristics or factors not examined in the present study. Therefore, future studies should aim to collect data from diverse populations while carefully considering differences in background characteristics.

## Supplementary Information

Additional file 1: Traffic light plot presenting the risk of bias judgments for each included study across all domains. Green, yellow, and red indicate low, unclear, and high risk of bias, respectively. The RoBANS 2 tool includes eight domains for assessing risk of bias: comparability of the target group (D1), target group selection (D2), confounders (D3), measurement of exposure (D4), blinding of assessors (D5), outcome assessment (D6), incomplete outcome data (D7), and selective outcome reporting (D8). Studies labeled with lowercase letters (a, b, c) indicate different subgroups (e.g., sex-specifi c cohorts) derived from the same original study. Summary plot showing the proportion of studies rated as low, unclear, or high risk of bias for each domain.

Additional file 2: The dotted line indicates the pooled effect size, and the shaded areas represent significance contours.

Additional file 3.

## Data Availability

The datasets and R scripts generated during the current study are available from the corresponding author upon reasonable request.

## Abbreviations

SMD: Standardized mean difference
LBM: Lean body mass
FFM: Fat-free mass
CSA: Muscle cross-sectional area
BIA: Bioelectrical impedance analysis
DEXA: Dual-energy X-ray absorptiometry
MRI: Magnetic resonance imaging
EUR: European
EAS: East Asian
AMR: Admixed American
